# Comparison of the physical activity levels between shift workers and non-shift workers in a large-scale cross-sectional study in Iran

**DOI:** 10.1186/s12889-023-16895-y

**Published:** 2023-10-18

**Authors:** Anahita Najafi, Roya Safari-Faramani, Maryam Selk-Ghaffari, Farid Najafi, Mohammad Ghafouri, Mitra Darbandi, Behnaz Mahdaviani, Amin Nakhostin-Ansari

**Affiliations:** 1 Sports Medicine Research Center, Neuroscience Institute, Tehran University of Medical Sciences, Tehran, Iran; 2 Research Center for Environmental Determinants of Health (RCEDH), Health Institute, Kermanshah University of Medical Sciences, Kermanshah, Iran

**Keywords:** Iran, Prevalence, Physical activity, Shift work

## Abstract

**Background:**

Shift work has been related to adverse health outcomes that can partially be attributed to physical inactivity. However, our knowledge of the influence of shift work on physical activity and sedentary behavior is inconclusive. Therefore, this study aimed to assess physical activity levels among shift and non-shift workers among a sample of Iranian adults.

**Methods:**

Baseline data of the Ravansar Non-Communicable Disease (RaNCD) cohort study were used. All participants of RaNCD except those excluded due to unemployment or considerable disability were included in the study. We evaluated participants’ physical activity levels using the PERSIAN cohort questionnaire and examined its associations with being a shift worker.

**Results:**

A total of 4695 participants with a mean age of 46.1 (SD = 7.74) were included in the study. In total, 1108 (23.6%) participants were shift workers, 1420 (30.2%) had insufficient physical activity levels, and 4283 (91.2%) were male. The prevalence of physical inactivity was significantly lower among shift workers compared to non-shift workers (21% vs. 33.1%, p < 0.001). Multiple backward stepwise binary logistic regression tests indicated that being a shift worker was significantly associated with a lower chance of having insufficient physical activity levels (OR = 0.77, 95% CI = 0.65–0.92, p = 0.003).

**Conclusions:**

The prevalence of insufficient physical activity was higher among non-shift workers than shift workers in our study. By providing the factors associated with insufficient physical activity among the workers in a region of Iran, the current study findings might help policymakers target groups at higher risk of physical activity in Iran and design interventions to improve physical activity, especially among non-shift workers.

## Introduction

Due to the modern economic changes and the rising societal demands for 24-hour services in recent decades, the number of shift workers has increased worldwide. In recent years, shift work accounts for about 20–25% of occupations. Providing services and operations round the clock and working-hour flexibility is beneficial for some people. However, emerging research has shown that late work shifts are associated with health-related adverse outcomes [1–4].

Night shift workers are often at risk of circadian rhythm disruptions. Circadian misalignment has been related to decreased sleep quality and quantity, increased stress, fatigue, physical discomfort, mental health problems, and burnout [5]. The irregular working hours may contribute to unhealthy habits, such as sedentary behavior, smoking, and unhealthy diets [6]. The psychological, physical, and behavioral effects of shift work may increase the chance of developing chronic illnesses. Studies have shown an increased risk of cancer, diabetes, hypertension, cardiovascular diseases, and gastrointestinal disorders among shift workers [7].

Adverse health outcomes among shift workers can partially be attributed to sedentary behavior and physical inactivity [8]. The effect of night shifts on physical activity levels has been widely investigated. However, the results are controversial. Some studies have reported an association between shift work and increased sedentary behavior [8, 9], whereas others have found that night shift workers are more physically active [10, 11]. Some studies found no significant association between shift work and physical activity levels [12]. A systematic review indicated that shift work is associated with spending less time on sedentary behavior, while there was no association between shift work and physical activity [13]. The inconsistency among findings suggests the need for more detailed investigations.

To our knowledge, no study in Iran compares the physical activity levels among shift and non-shift workers in Iran. Therefore, this study aimed to compare physical activity levels among shift and non-shift workers. Insights into the prevalence of sedentary behavior and physical activity and their contributing factors can shed light on possible health-promoting interventions for shift workers and help facilitate physical activity and promote healthy behavior.

## Methods

### Study design and population

This is a cross-sectional analysis conducted on the baseline data from the Ravansar Non-Communicable Disease (RaNCD) cohort study [14], which is one of the 18 study centers of the Prospective Epidemiological Research Studies in Iran (PERSIAN) cohort [15]. RaNCD is a prospective cohort study among Permanent residents of Ravansar district, western Iran, aged 35–65 years, and of Iranian Kurdish ethnicity. Baseline data were collected between March 2015 and February 2017. Written informed consent was obtained from all participants during the registration phase. Study design and protocol have been described previously [14]. The ethics committees of Kermanshah University of Medical Sciences (IR.KUMS.REC.1394.318) approved the study protocols.

### Participants

Data regarding the participants of the RaNCD cohort were retrieved and assessed for eligibility. Individuals who were unemployed or had a disability that prevented them from engaging in physical activity and those with incomplete data were excluded from the study. Recorded data of 10,047 individuals were assessed for eligibility. After excluding 5256 individuals due to unemployment and 96 cases due to disability, 4695 participants were included in the study.

### Study variables

Sociodemographic and lifestyle measures were recorded by interviewer-administered online questionnaires, as per the study’s protocols. Body composition measures were made upon visits to the cohort center using a bioelectric impedance machine (InBody 770, BIOSPACE KOREA).

#### Sociodemographic data

Participants were asked about their age at the time of the interview, gender (female/male), place of residence (urban or rural), marital status (single and ever married), educational status, smoking status, and current alcohol use. Participants were categorized into three groups of illiterates, those with an education level lower than a high school diploma (11 years of education), and those with a high school diploma or higher education levels. They were also categorized into non-smokers, current smokers, passive smokers, and former smokers regarding their smoking status.

#### Shift work

Participants were asked about working any night shifts in the past year and categorized into shift and non-shift workers based on their answers. Night shifts were defined as working for at least 6 h between 9 PM to 6 AM [15].

#### Socioeconomic status

Individuals’ socioeconomic status (SES) level index was derived using a principal component analysis (PCA) method. The SES relied on asset-related information such as the possession of various items like a freezer, laundry machine, dishwasher, personal computer with internet access, motorcycle, car, vacuum cleaner, and television in the household, as well as the availability of a cellphone, computer, laptop, internet access, and car (based on its value) for personal use and educational purposes, as well as the individual’s place of residence. Home ownership, area per capita, and room per capita are also considered for defining SES. SES groups were categorized into five quintiles, from the lowest (1st quintile) to the highest (5th quintile) [15–17].

#### Body mass index

The Body Mass Index (BMI, kg/m^2^) was calculated by dividing weight (kg) by height squared (m^2^). Participants were categorized into four groups based on their BMI: underweight (BMI < 18.5), normal BMI (18.5 ≤ BMI < 25), overweight (25 ≤ BMI < 30), and obese (30 ≤ BMI) [18].

#### Sleep duration

Participants were asked about their daytime sleep and sleep during the night. The total sleep duration of each participant was the sum of daytime and nighttime sleep duration. They were categorized into three groups based on their total sleep duration: short sleep duration (sleep duration ≤ 6 h), normal sleep duration (6 < sleep duration < 9), and long sleep duration (9 h ≤ sleep duration [19].

#### Physical activity

We used the Persian Cohort physical activity questionnaire to evaluate participants’ physical activity levels [15]. In the questionnaire, participants are asked about the average durations (in hours) of a number of activities on a normal day in the past year. We included activities with moderate and vigorous intensities (4 METs≤), including light technical jobs, masonry, light agricultural jobs, aerobic exercise, carrying light objects, heavy engineering jobs, heavy labor jobs, and heavy exercise in calculating physical activity levels for each participant [20, 21]. The time spent on repeated weekly or monthly activities was converted to daily times. Also, we only included activities in the analysis that participants had spent at least 10 min daily on them. The duration of each activity was multiplied by 60 to calculate the duration of the activity in minutes. Then, we multiplied it by 7 to calculate the duration of activity during a week. After that, the duration of activity was multiplied by its metabolic equivalent (MET). Total activity was calculated by summing the METs-min/week of activities with moderate to vigorous intensities. A total physical activity level of less than 600 METs-min/week was defined as having insufficient physical activity [22].

### Statistical analysis

Mean and standard deviation (SD) were used to describe quantitative variables, and frequency and percentage were used to describe categorical variables. We used Chi-square test to compare the categorical variables between groups. Also, we used the Kolmogorov-Smirnov test to evaluate the normal distribution of participants’ age. As it was not distributed normally (p < 0.05), we used the Mann-Whitney U test to compare participants’ ages between groups. We used multiple backward stepwise binary logistic regression tests to determine the factors independently associated with being physically inactive. We considered p < 0.05 statistically significant. All analyses were performed using SPSS version 26.

## Results

The basic and demographic characteristics of participants are presented in Table 1. The mean age of participants was 46.1 (SD = 7.74) years. In total, 1108 (23.6%) participants were shift workers, 1420 (30.2%) had insufficient physical activity levels, and 4283 (91.2%) were male. Among the shift workers, the median number of shifts of 10 (min = 1, max = 365, interquartile range = 27).

**Table 1:** Basic and demographic characteristics of participants (n = 4695)

Variable		Number (percentage)
Gender	Male	4283 (91.2%)
	Female	412 (8.8%)
Place of residence	Urban	2939 (62.6%)
	Rural	1756 (37.4%)
Marital status	Single	133 (2.8%)
	Ever married	4562 (97.2%)
Educational level	Illiterate	1260 (26.8%)
	Less than a high school diploma	2053 (43.7%)
	High school diploma or higher	1382 (29.4%)
Smoking	Never	1520 (32.8%)
	Current smoker	929 (20%)
	Former smoker	618 (13.3%)
	Passive smoker	1574 (33.9%)
Alcohol use	No	4237 (90.2%)
	Yes	458 (9.8%)
Body mass index (kg/m2)	Underweight (<18.4)	85 (1.8%)
	Normal (18.5–24.9)	1556 (33.4%)
	Overweight (25- 29.9)	2171 (46.6%)
	Obese (30≤)	847 (18.2%)
Socioeconomic status quantiles	First (worth)	464 (9.9%)
	Second	699 (14.9%)
	Third	906 (19.3%)
	Fourth	1150 (24.5%)
	Fifth (best)	1475 (31.4%)
Sleep duration	Short sleep duration (≤ 6 h)	568 (12.1%)
	Normal sleep duration (6-9 h)	3311 (70.5%)
	Long sleep duration (9 h≤)	816 (17.4%)

The physical activity status of different groups of participants is presented in Table 2. The prevalence of physical inactivity was significantly lower among shift workers compared to non-shift workers (21% vs. 33.1%, p < 0.001). Also, physical inactivity was more common among younger adults, females, those living in urban areas, single adults, those with higher educational levels, non-smokers, adults with obesity, and adults with better SES (p < 0.05).

**Table 2:** Prevalence of physical sufficient and insufficient physical activity among the participants based on their sociodemographic data

Variable		Insufficient physical activity (n = 1420)	Sufficient physical activity (n = 3275)	P-value	Chi-square value
Age (year)		45.24 (7.44)	46.47 (7.84)	***< 0.001***	-
Gender	Male	1103 (25.8%)	3180 (74.2%)	***< 0.001***	466.8
	Female	317 (76.9%)	95 (23.1%)		
Place of residence	Urban	1134 (38.6%)	1805 (61.4%)	***< 0.001***	259
	Rural	286 (16.3%)	1470 (83.7%)		
Marital status	Single	59 (44.4%)	74 (55.6%)	***< 0.001***	< 0.001
	Ever married	1361 (29.8%)	3201 (70.2%)		
Education status	Illiterate	266 (21.2%)	994 (78.9%)	***< 0.001***	147.6
	Less than a high school diploma	572 (27.9%)	1481 (72.1%)		
	High school diploma or higher	582 (42.1%)	800 (57.9%)		
Cigarrete smoking	Never	532 (35%)	988 (65%)	***< 0.001***	29.5
	Current smoker	243 (26.2%)	686 (73.8%)		
	Former smoker	160 (25.9%)	458 (74.1%)		
	Passive smoker	466 (29.6%)	1108 (70.4%)		
Alcohol use	No	1296 (30.6%)	2941 (69.4%)	0.12	2.4
	Yes	124 (27.1%)	334 (72.9%)		
Body mass index (kg/m2)	Underweight (18.4<)	16 (18.8%)	69 (81.2%)	***< 0.001***	68.5
	Normal (18.5–24.9)	379 (24.4%)	1177 (75.6%)		
	Overweight (25- 29.9)	680 (31.3%)	1491 (68.7%)		
	Obese (30≤)	337 (39.8%)	510 (60.2%)		
Socioeconomic status quantiles	First (worth)	64 (13.8%)	400 (86.2%)	***< 0.001***	243.2
	Second	125 (17.9%)	574 (82.1%)		
	Third	220 (24.3%)	686 (75.7%)		
	Fourth	375 (32.6%)	775 (67.4%)		
	Fifth (best)	635 (43.1%)	840 (56.9%)		
Sleep duration	Short sleep duration (≤ 6 h)	155 (27.3%)	413 (73.7%)	0.209	3.1
	Normal sleep duration (6-9 h)	1007 (30.4%)	2304 (69.6%)		
	Long sleep duration (9 h≤)	258 (31.6%)	558 (68.4%)		
Shift work	No	1187 (33.1%)	2400 (66.9%)	***< 0.001***	58.4
	Yes	233 (21%)	875 (79%)		

Values are reported as number (%), except for age, which is presented as mean (SD)

Table 3 presents the results of multiple backward stepwise binary logistic regression tests determining the factors independently associated with having insufficient physical activity levels. Being a shift worker was significantly associated with a lower chance of having insufficient physical activity levels (OR = 0.77, 95% CI = 0.65–0.92, p = 0.003).

**Table 3:** Results of multiple backward stepwise binary logistic regression tests to determine the factors independently associated with insufficient physical inactivity

Variable		Odds ratio	95% confidence interval	P-value
Gender	Male (reference)	*	*	*
	Female	9.05	7- 11.69	< 0.001
Place of residence	Urban (reference)	*	*	*
	Rural	0.47	0.4–0.56	< 0.001
Educational level	Less than high school diploma (reference)	*	*	*
	High school diploma or higher degrees	1.37	1.16–1.62	< 0.001
Body mass index	Normal or underweight (reference)	*	*	*
	Overweight or obese	1.23	1.06–1.43	0.004
Sleep duration	Short or normal sleep duration (< 9 h) (reference)	*	*	*
	Long sleep duration (9 h≤)	1.31	1.09–1.57	0.004
Socioeconomic status	First quantile (reference)	*	*	*
	Second quantile	1.4	0.99- 2	0.061
	Third quantile	1.95	1.4–2.72	< 0.001
	Fourth quantile	2.47	1.78–3.41	< 0.001
	Fifth quantile	3.13	2.23–4.39	< 0.001
Shift work	No (reference)	*	*	*
	Yes	0.77	0.65–0.92	0.003

## Discussion

The present study investigated physical activity levels and their associated factors among a large sample of adults in Ravansar province, Iran. Our results demonstrated that 30.2% of the studied population and 21% of shift workers do not engage in sufficient physical activity. This finding suggests suitable interventional strategies should be designed based on work schedules and contributing factors. However, the prevalence of insufficient physical activity was lower among shift workers than among non-shift workers. Also, insufficient physical activity was associated with the female gender, living in urban areas, having higher educational levels, having higher BMIs, long sleep duration, and better SES.

Insufficient physical activity prevalence in our studied population is consistent with the standardized global and regional prevalence, which were reported to be 27.5% and 32.8%, respectively [23]. However, it must be noted that in female participants, insufficient physical activity prevalence was 76.9%, which is considerably higher than the global and regional rates (31.7% globally and 39.9% in the Middle East region), highlighting the need for interventional policies to facilitate physical activity in women [23]. Participants in the current study all had jobs, and it seems that female workers, regardless of being shift workers or non-shift workers, might be a group in Iran with a higher risk of insufficient physical activity and its consequences. Therefore, considering the efficacy of workplace interventions to increase physical activity among females [24], our finding emphasizes the necessity of implementing such interventions for Iranian female workers.

Previous studies have investigated physical activity levels among shift and non-shift workers and have found mixed results. While some studies found that shift workers were more physically active than non-shift workers, others reported shift workers as less active than non-shift workers. Hulsegge et al. investigated a large number of individuals working in the Netherlands’ industrial production. They reported that shift workers were more engaged in physical activities than non-shift workers [25]. In contrast, a study conducted on a database of US civilians of various occupations reported that individuals working night or evening shifts engaged in less sustained physical activity. However, the total amount of time engaged in moderate to vigorous physical activity did not differ among groups [8]. In a survey conducted in Australia on individuals working in various sectors, Vandelanotte et al. reported that shift workers engaged in less leisure-time physical activity and more occupational physical activity compared to non-shift workers. The overall physical activity level was slightly higher in shift workers [26]. In another study, physical activity levels among hospital workers were objectively measured. Hospital shift workers spent more time walking during working hours and less time in sedentary behavior. However, the two groups had no significant difference concerning overall physical activity levels [27]. Van de Langenberg et al. investigated physical activity levels in shift and daytime hospital workers using self-report and objective measures. While objectively measured physical activity levels did not differ among the two groups, night shift workers’ self-reported physical activity levels were significantly higher [28]. Monnaatsie et al. implemented a systematic review and meta-analysis on physical activity and sedentary behavior in shift and non-shift workers. They found no significant associations between shift work and physical activity levels. On the other hand, pooled data of self-report and objective measures showed that shift workers spent less time in sedentary behavior [13].

The aforementioned findings do not provide strong evidence of the association between shift work and physical activity levels. The discrepancy between previous studies and our results could be attributed to different measurement tools since self-report assessment tools may result in recall bias compared to measurement devices [28, 29]. Additionally, the observed discrepancies may have contributed to environmental and cultural differences and physical demands of multiple types of shift work. In shift work occupations, occupational activity may account for a greater portion of the individuals’ total physical activity compared to leisure-time activity [11, 26]. Although any physical activity is beneficial and recommended [30], the role of occupational activity on health outcomes is inconclusive. Workers may not benefit from the increased physical activity due to occupational demand compared to the more sustained activities during leisure time [31, 32].

Overall, it seems that based on the cultural and work situation of each region and country, shift workers might engage in more or less physical activity compared to non-shift workers. Therefore, countries should study such an association in their own context instead of relying on previous studies in other world regions. In our study, a possible explanation for shift workers’ higher physical activity levels might be that a higher proportion of shift workers, compared to non-shift workers, lived in rural areas. Working in rural areas and engaging in agricultural activities might be a possible reason for higher physical activity among shift workers in our study. On the other hand, urbanization and lifestyle changes in urban areas might be another reason for lower physical activity levels among the non-shift workers in our study [33].

### Limitation

Our study’s strength lies in employing a large sample of the Iranian population and examining numerous covariates comprehensively. However, the participants were enrolled from a single region, limiting the generalizability of our findings to the whole nation or other parts of the world. The cross-sectional design, unknown occupation types, and self-report measurements of physical activity are other limitations of our study. Future multicenter studies, with a longitudinal design and utilization of objective measures of physical activity, might be helpful in providing policymakers with better insight regarding the association between shift work and physical activity. Furthermore, we asked participants about their physical activity levels in the past year prior to the study, which might be subject to recall bias. We also used the PERSIAN cohort questionnaire to assess physical activity, which is not one of the commonly used questionnaires, such as global physical activity questionnaire or International Physical Activity Questionnaire, to evaluate physical activity. While incorporating this questionnaire will facilitate comparing our results with those from different regions of Iran, it’s important to consider that this approach might limit the extent to which our findings can be directly compared to results from other countries. Finally, only 8.8% of our study participants were females, which may reflect the workforce distribution in Iran, where the number of employed males tends to be higher than that of employed females [34]. However, the small number of females in our study may reduce the generalizability of our findings for female workers in Iran.

## Conclusion

The prevalence of insufficient physical activity was higher among non-shift workers compared to shift workers in our study. By providing the factors associated with insufficient physical activity among the workers in a region of Iran, the current study’s findings might help policymakers target groups at higher risk of physical activity in Iran and design interventions to improve physical activity, especially among the non-shift workers.

## Data Availability

The datasets generated and/or analyzed during the current study are not publicly available according to the PERSIAN cohort guidelines but are available from the corresponding author on reasonable request.

## List of abbreviations

BMI: Body mass index
MET: Metabolic equivalent
PERSIAN: Prospective Epidemiological Research Studies in Iran
RaNCD: Ravansar Non-Communicable Disease
SD: Standard deviation
SES: Socioeconomic status
